# Methane alleviates sepsis-induced injury by inhibiting pyroptosis and apoptosis: in vivo and in vitro experiments

**DOI:** 10.18632/aging.101831

**Published:** 2019-02-18

**Authors:** Zeyu Li, Yifan Jia, Yang Feng, Ruixia Cui, Runchen Miao, Xing Zhang, Kai Qu, Chang Liu, Jingyao Zhang

**Affiliations:** 1 Department of Hepatobiliary Surgery, The First Affiliated Hospital of Xi’an Jiaotong University, Xi’an Shaanxi 710061, People's Republic of China; 2 Department of Immunology, Shaanxi University of Chinese Medicine, Xianyang Shaanxi 712046, People's Republic of China; 3 Department of ICU, The First Affiliated Hospital of Xi’an Jiaotong University, Xi’an Shaanxi 710061, People's Republic of China; 4 Department of SICU, The First Affiliated Hospital of Xi’an Jiaotong University, Xi’an Shaanxi 710061, People's Republic of China; * Equal contribution

**Keywords:** methane, sepsis, anti-pyroptosis, anti-inflammation, anti-apoptosis

## Abstract

Sepsis is defined as a life-threatening organ dysfunction caused by a dysregulated host response to infection. Methane has been reported to have anti-oxidative, anti-apoptotic and anti-inﬂammatory properties. We investigated the potential protective effects of methane on sepsis-induced injury and determined the related mechanisms. C57BL/6 mice received laparotomy with cecal ligation and puncture (CLP) to create a septic model, followed by methane-rich saline (MRS) treatment after CLP. MRS treatment improved the 5-day survival rate and organ functions and alleviated pathological damage of the mice, as well as reduced excessive inflammatory mediators, such as tumor necrosis factor-α and interleukin-6. MRS treatment also decreased the levels of oxidative stress index proteins, decreased the apoptosis of cells and inhibited nod-liker receptor protein (NLRP)3-mediated pyroptosis in the lung and intestine. In in vitro experiments, RAW264.7 and primary peritoneal macrophages were treated with lipopolysaccharide (LPS) plus adenosine-triphosphate (ATP) to induce inflammation and pyroptosis. Consistent with the in vivo results, methane-rich medium (MRM) treatment also reduced the levels of excessive inflammatory mediators, and decreased the levels of ROS, inhibited apoptosis and pyroptosis. Our results indicate that methane offers a protective effect for septic mice via its anti-inflammation, anti-oxidation, anti-pyroptosis and anti-apoptosis properties.

## Introduction

Sepsis, a systemic inflammatory response syndrome caused by polymicrobial infections, is a major cause of mortality in hospital patients [1]. An excessive, active, innate immune response has been considered the primary underlying cause of septic damage, leading to imbalances in host homeostasis, endothelial dysfunction, and metabolism due to parenchymal cellular maladjustment [2]. During sepsis, the activation of Kupffer cells can activate the nuclear factor-kappa B (NF-κB) signaling pathway, which further accelerates the production of proinflammatory cytokines, such as tumor necrosis factor-α (TNF-α), interleukin-6 (IL-6) and interleukin-1β (IL-1β), which form the cytokine cascade, and eventually leads to cell apoptosis and multiple organ dysfunction [3,4]. In addition, NF-κB further stimulates the production of pro-IL-1β, which plays key roles in the NOD-like receptor protein 3 (NLRP3)-mediated pyroptosis. Pyroptosis is a kind of inflammasome pathway-mediated programmed cell death. During pyroptosis, many 1-2 nm pores are formed on the cell membrane, causing the cell membrane to lose integrity and the ability to import and export control material, eventually leading to the dissolution of the cell membrane, the release of cellular contents, and further induction of an inflammatory response in other cells [5]. NLRP3, a multi-protein complex, is a type of inflammasome that, can recognize pathogen associated molecular patterns (PAMPs) and execute inflammatory responses [6]. Active NLRP3 promotes the secretion of the cytokines IL-1β and the cysteine proteases (caspase-1), which are considered protective during initial process of sepsis. However, when the proinflammatory cytokines, such as IL-1β, are continually released, tissue injury and organs dysfunction can occur [7]. Excessive reactive oxidative species (ROS) produced by inflammatory reactions also cause activation of NLRP3, which indicates that ROS also can induce pyroptosis-induced multiple organ dysfunction [8]. Therefore, the pharmacological inhibition of the NLRP3 pathway might constitute a potent protective strategy during sepsis.

Methane, one of the most abundant organic and greenhouse gases found in the atmosphere [9], is a biologically inactive gas that has gained increasing attention, especially as a potential disease treatment. Recent studies have revealed that methane has a positive effect on sepsis-induced liver injury via its anti-oxidation, anti-inflammation and anti-apoptotic properties [10]. Methane-rich saline (MRS) also can ameliorates sepsis-induced acute kidney injury via regulating endoplasmic reticulum stress (ERS) [11]. ERS is also related to the activation of NLRP3 [12]. We therefore hypothesized that MRS alleviates sepsis-induced multiple organ dysfunction via inhibiting excessive inflammatory responses and decreasing oxidative stress, thereby reducing cell apoptosis and pyroptosis. In this study, MRS was prepared and used to explore its therapeutic effects in septic mice and to investigate its underlying specific mechanisms.

## RESULTS

### MRS alleviates tissue damage and improves the survival rate

**Figure 1:**
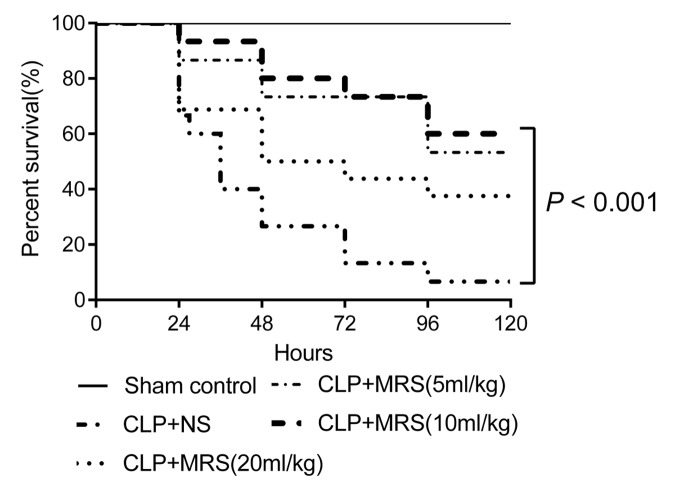
**Methane-rich saline improved the survival ratio of mice.** Kaplan-Meier survival curve for mice 5 days after the cecal ligation and puncture (CLP) operation (n = 9). Sham control group received 2.5ml/kg normal saline every 12 h; Normal saline (NS) group received 2.5ml/kg normal saline every 12 h; Methane-rich saline (MRS) group (5ml/kg) received 5mL/kg MRS every 12 h; MRS group (10ml/kg) received 10mL/kg MRS every 12 h; MRS group (20ml/kg) received 20mL/kg MRS every 12 h.

To examine the protective effect of MRS during sepsis-induced injury, the mice were randomized and monitored to evaluate their 5-day survival rates after receiving different concentration of MRS every 12 h. Log-rank test analysis of the 5-day survival curves for CLP-induced sepsis demonstrated that the survival rates of the CLP+MRS groups were significantly higher than those of the CLP+NS group (Figure 1). The survival rates of the CLP+MRS groups varied with the different concentrations of MRS. The survival rate of the 10 ml/kg CLP+MRS group was significantly higher than that of the 5 ml/kg and 20 ml/kg CLP+MRS groups (*P* < 0.001). Therefore, we used 10 ml/kg MRS as the optimal therapeutic concentration for subsequent experiments.

**Figure 2:**
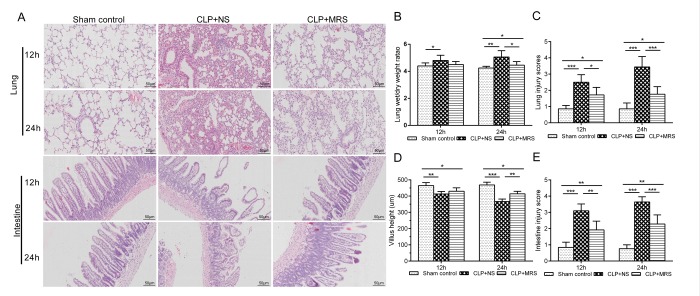
**Methane-rich saline alleviated the histopathology damage and organ dysfunction induced by sepsis. **The lung and intestine tissues were collected 12 and 24 h after the cecal ligation and puncture (CLP) operation. (**A**)** R**epresentative hematoxylin and eosin (H&E) staining of lung and intestine sections (Scale bars: 50μm). (**B**) The lung wet/dry weight ratio was calculated as an assessment of lung injury. (**C**) Lung injury scores. (**D**) The villus height was measured as an assessment of intestine injury. (**E**) Intestine injury scores. (n = 12. Data are shown as the mean ± SD. ^*^P < 0.05;^ **^p < 0.01; ^***^P < 0.001).

Next, we used HE staining to evaluate the protective effect of MRS on the lungs and intestines 12 and 24 h after CLP (Figure 2A). The CLP+NS group showed markedly increased levels of congestion, inflammatory cell infiltration, necrosis, and degeneration in the lung and intestine compared with those in the CLP+MRS group. Additionally, the lung W/D weight ratio and lung injury scores were increased markedly at 12 and 24 h after CLP in the CLP+NS group compared with those in the Sham control group. The administration of MRS significantly reduced the lung W/D weight ratio and lung injury scores 12 and 24 h after CLP (*P* < 0.001) (Figure 2B-C). The CLP+NS group mice were marked decreased in villus height and showed increased intestine injury scores 12 and 24 h after CLP (Figure 2D-E). MRS treatment alleviated the sepsis-induced intestine injury, and the CLP+MRS group showed significantly higher villus heights and lower intestine injury scores than those in the CLP+NS group 12 and 24 h after CLP (*P* < 0.01). Based on these results above, we concluded that MRS treatment improves the survival rate and alleviates tissue damage in septic mice.

### MRS downregulates inflammatory responses

To determine whether MRS treatment can decrease the inflammatory response, the levels of TNF-α and IL-6 were measured in the septic mouse blood samples and RAW 264.7 and primary peritoneal macrophage supernatant. In the in vivo experiment, the levels of TNF-α and IL-6 were significantly increased in the CLP+NS group 12 and 24 h after CLP compared with those in the Sham control group (*P* < 0.001). MRS treatment significantly decreased the levels of inflammatory indicators at 12 and 24 h (*P* < 0.01) (Figure 3A-B). Consistent with the in vivo experiment, the levels of TNF-α and IL-6 in both RAW 264.7 and primary peritoneal macrophages were significantly increased after stimulation with LPS+ATP compared with those in the NM and MRM groups (Figure 3C-D). The NM+L+A group showed higher levels of TNF-α and IL-6 than those of the MRM+L+A group (*P* < 0.05), indicating that MRM could reduce the excessive secretion of TNF-α and IL-6 induced by CLP/LPS+ATP. According to these results, we found that methane treatment could decrease the inflammatory responses induced by sepsis.

**Figure:**
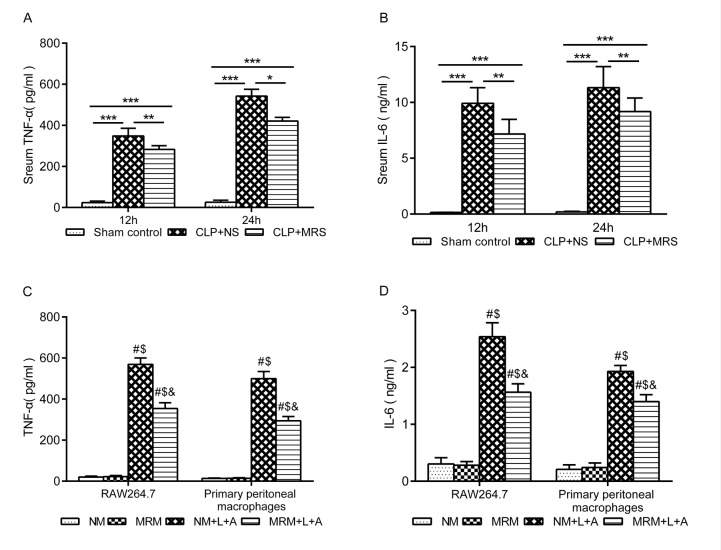
** Figure 3. Methane suppressed activation of inflammatory cytokine. **The blood samples were collected 12 and 24 h after the cecal ligation and puncture (CLP) (n = 12). The levels of serum (**A**) tumor necrosis factor (TNF)-α and (**B**) interleukin (IL)-6 were detected by ELISA. The supernatant of RAW 264.7 cells and primary peritoneal macrophages was collected 24 h after lipopolysaccharide+ adenosine-triphosphate (LPS + ATP) stimulation (n = 3). The levels of (**C**) TNF-α and (**D**) IL-6 in supernatant were detected by ELISA. (Data are shown as the mean ±SD. ^*^P < 0.05;^ **^p < 0.01; ^***^P < 0.001; ^#^*P* < 0.05 versus normal medium (NM) group;^ $^*p* < 0.05 versus methane-rich medium (MRM) group; ^&^*P *< 0.05 versus. normal medium+ lipopolysaccharide+ adenosine-triphosphate (NM+L+A) group).

### MRS reduces oxidative stress in sepsis

Oxidative stress is an initial and crucial factor in the process of sepsis injury [13]. To further determine whether the anti-oxidative property of MRS plays a key role in the protective effect of MRS, we measured the expression levels of ROS indicators in the lung and intestine 12 and 24 h after CLP. The levels of MPO and MDA in the lung and intestine presented an increased rising trend compared with those in the Sham control group, especially for the CLP+NS group, in which the levels increased to a greater extent than those in the CLP+MRS group (P < 0.05) (Figure 4A-D). The levels of GSH and SOD in the lung and intestine tissue gradually were decreased in the CLP+NS group compared with those in the Sham control group, and this decrease was reversed by MRS treatment (*P* < 0.05) (Figure 4E-H). Additionally, in the in vitro experiment, DHE fluorescence for ROS detection revealed a marked increase in the fluorescence intensity after LPS+ATP stimulation (Figure 4I-J). MRM treatment significantly decreased the fluorescence intensity compared with that in the NM+L+A group (*P* < 0.05). These results suggested that methane treatment could reduce sepsis-induced oxidative stress to prevent tissue injury.

**Figure:**
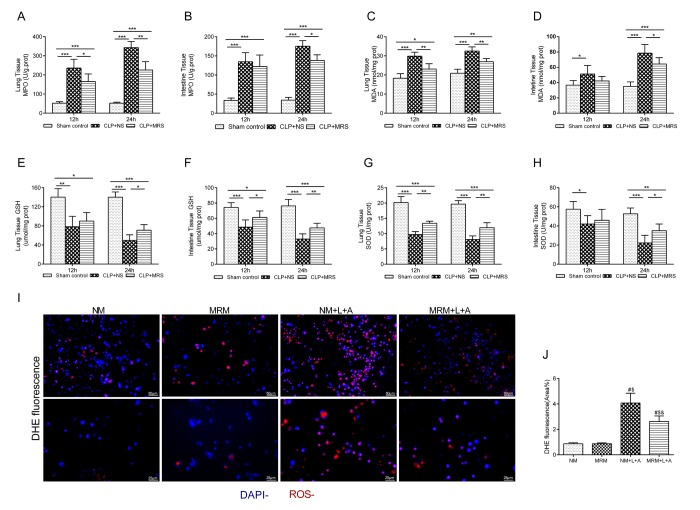
** Figure 4. Eﬀects of methane on antioxidants and oxidative products. **The lung and intestine tissues were harvested 12 and 24 h after cecal ligation and puncture (CLP) (n = 12). The (**A**-**B**) Myeloperoxidase (MPO), (**C**-**D**) malonaldehyde (MDA), (**E**-**F**) glutathione (GSH) and (**G**-**H**) superoxide dismutase (SOD) in lung and intestine were detected 12 and 24h after CLP. (**I**–**J**) Representative dihydroethidium (DHE) fluorescence staining in RAW 264.7 cells and the calculated fluorescence intensity (Scale bars: 25 μm and 50 μm) (n = 3). (Data are shown as the mean ±SD. ^*^P < 0.05;^ **^p < 0.01; ^***^P < 0.001; ^#^*P* < 0.05 versus normal medium (NM) group;^ $^*p* < 0.05 versus methane-rich medium (MRM) group; ^&^*P *< 0.05 versus. normal medium+ lipopolysaccharide+ adenosine-triphosphate (NM+L+A) group).

### MRS exerts anti-apoptotic effects on pulmonary cells, intestinal epithelial cells after sepsis

To determine whether MRS could also inhibit apoptosis that occurs in multiple organs, we performed TUNEL staining. The TUNEL-positive cells corresponded to the distribution of pulmonary cells and intestinal epithelial cells (Figure 5A). Few apoptotic cells were discovered in the Sham control groups. However, 24 h after CLP, abundant TUNEL-positive cells in the lung and intestine were identified in the CLP+NS groups (p < 0.05). By contrast, fewer apoptotic cells were identified in these two organs in the CLP+MRS groups (p < 0.05). The quantification of apoptotic pulmonary cells and intestinal epithelial cells verified these findings (Figure 5B). We further used Western blot analysis of activated cytochrome c, caspase-3 and caspase-9 in the lung and RAW 264.7 and primary peritoneal macrophages to detect how MRS affects the apoptotic process. The gray-scale ratios of the CLP+MRS group were reduced significantly compared with those of the CLP+NS group, matching the TUNEL results (Figure 5C-F). Methane attenuated these apoptotic eﬀects (p < 0.05). Additionally, in the in vitro experiment, flow cytometry analysis indicated that inflammation accelerated the massive apoptosis of RAW 264.7 cells after stimulation with LPS+ATP (Figure 5G-H). However, significant reductions in the apoptotic cell percentage were observed with MRM treatment, 24 h after stimulation with LPS+ATP. According to these results above, we concluded that methane treatment can interfere with cell death to offer a protective effect.

**Figure:**
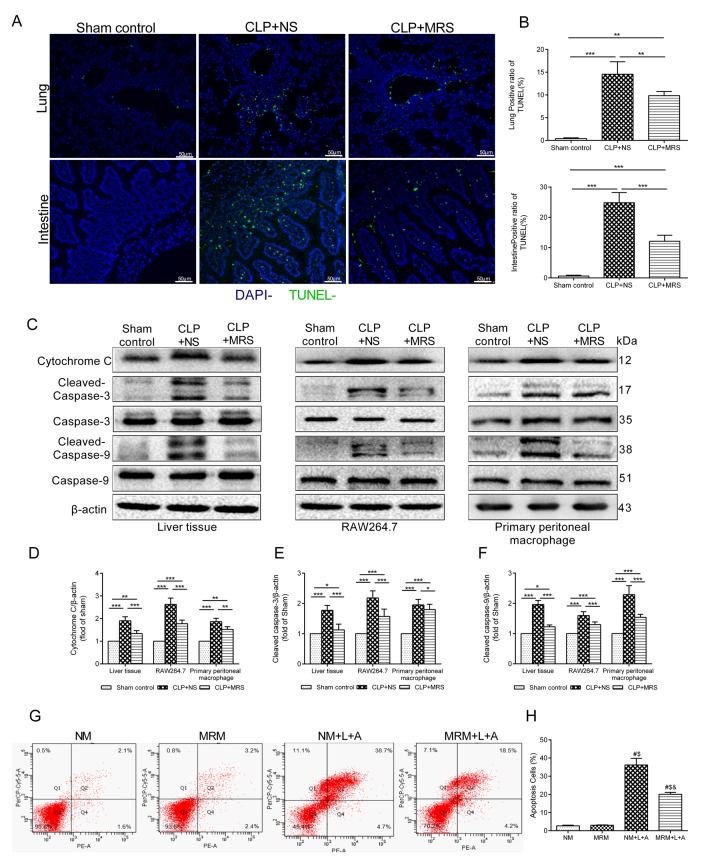
** Figure 5. Methane reduced apoptosis induced by sepsis. **The lung and intestine tissues were harvested 12 and 24h after cecal ligation and puncture (CLP) (n = 12). (**A**) Representative TUNEL staining in lung and intestine tissues (Scale bars: 50μm). (**B**) The percentage of TUNEL positive cells were measured. (**C**) Representative Western blot and quantitative analysis of (**D**) Cytochrome c, (**E**) caspase-3, (**F**) caspse-9 in lung tissue, RAW264.7 cells and primary peritoneal macrophages. (**G**-**H**) Flow cytometry analysis of RAW264.7 cell apoptosis percentage at 24 h after the stimulation of lipopolysaccharide+ adenosine-triphosphate (LPS+ATP) (n = 3). (Data are shown as the mean ±SD. ^*^P < 0.05;^ **^p < 0.01; ^***^P < 0.001; ^#^*P* < 0.05 versus normal medium (NM) group;^ $^*p* < 0.05 versus methane-rich medium (MRM) group; ^&^*P *< 0.05 versus. normal medium+ lipopolysaccharide+ adenosine-triphosphate (NM+L+A) group).

### MRS treatment inhibits pyroptosis

**Figure 6:**
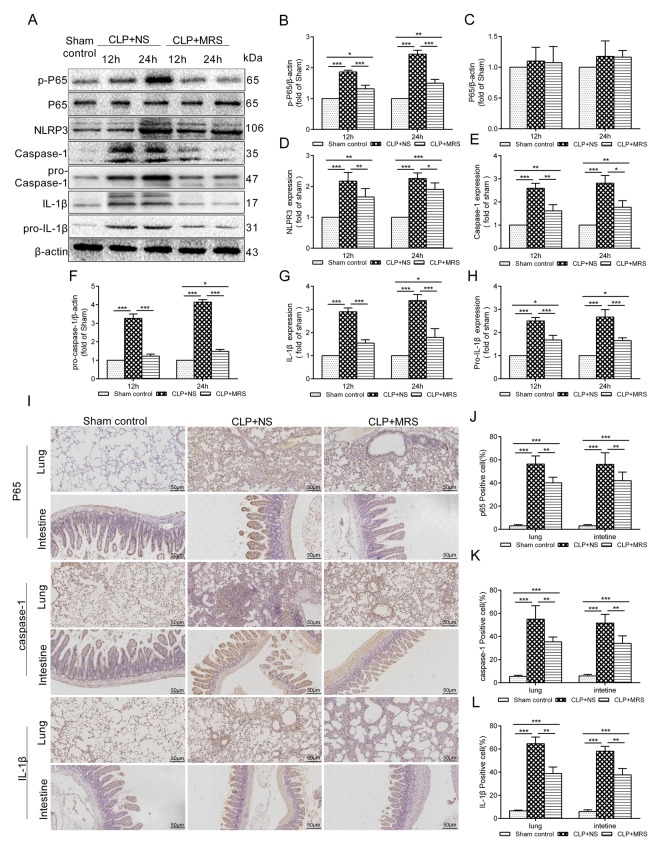
**Methane-rich saline downregulated the NLRP3/Caspase-1/IL-18, IL-1β signaling pathway.** The lung tissues were harvested 12 and 24 h after cecal ligation and puncture (CLP). (**A**) Representative immunoblots of p-p65, p65, pro-Caspase-1, pro-IL-1β and NLRP3/Caspase-1/ IL-1βfrom lung tissues 12 and 24h after MRS treatment. Relative densities of (**B**) p-P65. (**C**) P65. (**D**) NLRP3. (**E**) caspase-1. (**F**) pro-caspase-1. (**G**) IL-1β. (**H**) pro-IL-1β were calculated. (**I**) Representative immumohistochemical staining of P65, caspse-1 and IL-1β in the lung and intestine tissues. (**J**-**L**) The percentage of P65, caspse-1 and IL-1β positive cells were calculated. (Scale bars: 50μm. Data are shown as the mean ±SD. ^*^P < 0.05;^ **^p < 0.01; ^***^P < 0.001).****

To explore whether MRS could affect the NLRP3 inflammasome signaling pathway to improve sepsis injury, we measured the expression levels of p-P65, P65, pro-caspase-1, pro-IL-1β and NLRP3/caspase-1/IL-1β by Western blotting 12 and 24 h after CLP. The results showed that MRS treatment significantly reduced the expression levels of p-P65, P65, pro-caspase-1, pro-IL-1β and NLRP3/caspase-1/IL-1β compared with those of the CLP+NS group, confirming that MRS could reduce pyroptosis and protect tissue and organs (Figure 6A). Quantification of the relative protein expression levels verified these findings (P < 0.05) (Figure 6B-H). Additionally, lung and intestinal tissues were collected 24 h after CLP and the expression levels of P65, caspase-1 and IL-1β were detected by immunohistochemistry staining (Figure 6I-L). MRS treatment could effectively reduce the expression levels of P65, caspase-1 and IL-1β in tissues compared with those of the CLP+NS group (*P* < 0.05), consistent with the previous results. In an in vitro experiment, LPS+ATP was used to induce pyroptosis and inflammation. Propidium iodide (PI) staining was used to detect pyroptosis. MRM treatment significantly inhibited pyroptosis induced by LPS+ATP compared with that of the NM+L+A group (*P* < 0.05) (Figure 7A-B). MRM treatment also significantly inhibited the expression of p-P65, P65, pro-caspase-1, pro-IL-1β and NLRP3/caspase-1/ IL-1β after the induction of LPS+ATP (Figure 7C-G), consistent with PI staining. IL-1β immunostaining was increased markedly 24 h after LPS+ATP stimulation in RAW 264.7 cells. The administration of MRM significantly reduced the IL-1β-positive cells (*P* < 0.05) (Figure 7H-I). Based on these results, we concluded that methane treatment could inhibit pyroptosis by downregulating the expression of levels of proteins related to the inflammasome signaling pathway, which decreased septic injury.

**Figure:**
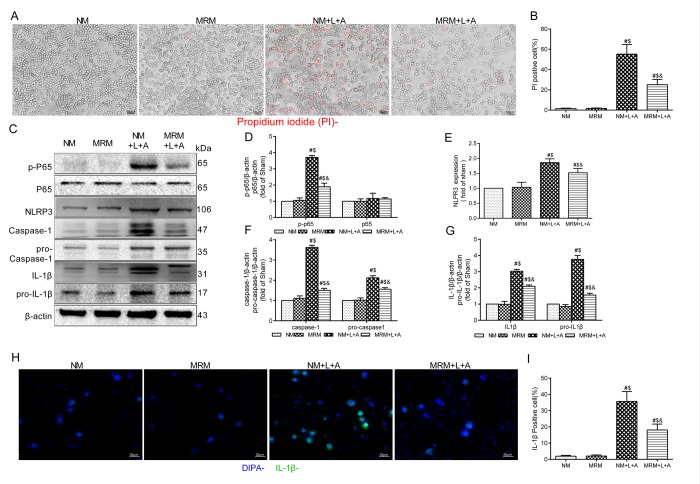
** Figure 7. Methane restrained pyroptosis in vitro. **(**A**) Representative propidium iodide (PI) staining of RAW 264.7 cells (Scale bars: 25μm). (**B**) The percentage of PI positive cells. (**C**) Representative immunoblots of p65, p-p65, pro-caspase1, pro-IL-1β and NLRP3/Caspase-1/IL-1βfrom RAW264.7 cells. (**D**) Relative densities of p-P65 and P65, (**E**) NLRP3, (**F**) caspase-1 and pro-Caspase-1, and (**G**) IL-1β and pro-IL-1β were calculated. (**H**) Representative immunofluorescence staining of IL-1β (Scale bars: 25μm). (**I**) The percentage of IL-1β positive cells was calculated. (n = 3. Data are shown as the mean ±SD. ^#^*P* < 0.05 versus NM (normal medium) group;^ $^*p* < 0.05 versus MRM (methane-rich medium) group; ^&^*P *< 0.05 versus. NM+L+A (normal medium+ lipopolysaccharide+ adenosine-triphosphate) group).

## DISCUSSION

Sepsis is a clinical syndrome characterized by an incongruous host response and accompanied by multiple organ dysfunction. The mechanism of sepsis is complicated, involving excessively expressed cytokines, ROS and apoptosis [14]. In the present study, we showed that treatment with MRS alleviated sepsis-induced injury in mice. The potential mechanisms might be related to MRS’s role in inhibiting excessive inflammatory responses, decreasing oxidative stress damage and thereby reducing apoptosis and pyroptosis. Our research is the first study to comprehensively explore the protective effects of MRS on sepsis, both in vivo and in vitro and to demonstrate that MRS may offer a novel therapeutic approach to sepsis.

Methane, one of the most abundant and simplest organic gases in the world, is important for the greenhouse effect. Methane and hydrogen gas are the products of bacterial metabolism in the human gut, while hydrogen can also be converted to methane in the gut by some methane-producing bacteria [15]. However, the physiological effects of methane in the human metabolic process have been neglected for a long time, and the function of methane during disease treatment remains poorly understood. As one of the simplest aliphatic hydrocarbons, methane has been increasingly studied for its medical applications. With its lipid-soluble peculiarity, methane can easily cross the cell membrane and persist in tissues, another reason why methane could be used as a novel method to treat sepsis [16]. Methane possesses anti-oxidative, anti-inflammatory, and anti-apoptotic activities, which have been demonstrated through a rat model [17]. However, studies on the effects of methane on organ dysfunction induced by sepsis remain scarce.

In this study, we demonstrated the protective effect of MRS on sepsis in both in vivo and in vitro experiments. MRS treatment improved the 5-day survival rate of mice after CLP compared with that of the CLP+NS group. We also discovered that the mice that underwent CLP exhibited multiple organ dysfunction, including the lung and intestine, which were characterized by histopathologic changes, inflammatory injury, oxidative damage and cell apoptosis. MRS treatment protected the host from multiple organ dysfunction induced by sepsis. Additionally, the excessive expression of inflammatory cytokines (TNF-α and IL-6) during sepsis leads to tissue and cell damage. MRS treatment significantly decreased the expression levels of proinflammatory cytokines to provide a protective effect. Moreover, MRS treatment decreased the expression of MPO and MDA, and increased the levels of GSH and SOD, thereby reducing oxidative stress injury during sepsis. Additionally, MRS treatment downregulated the expression of cytochrome c, caspase-3 and caspase-9, reduced apoptosis and alleviated sepsis damage. Finally, MRS suppressed the activation of the NLRP3/Caspase-1/IL-1β inflammasome signaling pathway to alleviate the tissue and cell damage induced by sepsis.

Inflammation plays an important role in the pathogenesis of sepsis. Bacteria and their metabolic products can trigger an uncontrollable inflammatory response in a host, causing the host cells to release many inflammatory mediators, including IL-6 and TNF-α. Those overproduced inflammatory mediators further influence the progress of sepsis and induce systemic inflammatory responses and organ damage,leading to death [18]. Our study demonstrated that the levels of TNF-α and IL-6 exhibited a significant upward trend after CLP/LPS+ATP compared with those in the Sham group. However, this upward trend was reversed by MRS/MRM treatment, indicating that methane could reduce the levels of TNF-α and IL-6 to offer a protective effect for tissues and organs during sepsis.

Oxidative stress caused by sepsis also induces tissue and cell damage [19]. The proliferation of ROS induced by sepsis leads to an unbearable state of anti-oxidant defense, directly causing mitochondrial damage, a key event in sepsis-induced organ dysfunction [20]. SOD and GSH are low- molecular-weight molecules and are critical enzymes in anti-oxidant defense, which can scavenge ROS generated from H_2_O_2_ and oxygen. MDA and MPO are markers of oxidative stress in damaged lipids. In our study, the MPO and MDA levels increased significantly after CLP, while the SOD and GSH levels decreased significantly. These tendencies were mitigated by MRS treatment, indicating that MRS protected the host from oxidative stress injury through regulating the expression of ROS products.

Sepsis causes multiple organ dysfunction partly because of tissue cell apoptosis [21]. Sepsis leads to the unbalanced metabolism of oxygen, which induces damage to the mitochondrial membrane, releasing cytochrome c, activating caspase-3, and ultimately causing apoptosis [22]. According to our work, the number of TUNEL-positive cells significantly increased in lung and intestine tissues 24 h after CLP. MRS treatment alleviated the rate of TUNEL positive cells compared with that in the CLP+NS group. Meanwhile, the activation levels of cytochrome c, caspase-3 and caspase-9 significantly increased after CLP/LPS+ATP compared with those in the Sham control group, but MRS/MRM treatment could inhibit the activation of caspase-3 and caspase-9. Additionally, flow cytometry analysis indicated that MRM treatment reduced cell apoptosis in the in vitro experiment. Thus, we concluded that methane may prevent tissue cell apoptosis to provide a protective function.

Infection and ROS activate NF-κB to mediate the expression of NLRP3 and increase the production of pro-IL-1β. The NLRP3 inflammasome is a multiprotein complex that activates caspase-1 during infection and ROS. The activation of caspase-1 further drives inflammation through the conversion of pro-IL-1β to IL-1β, subsequently inducing cell death [23]. Our study demonstrated that the activation levels of NF-κB (p65), pro-caspase-1, pro-IL-1β, NLRP3, caspase-1 and IL-1β were increased after CLP/LPS+ATP, illustrating that the NLRP3/caspase-1/IL-1β signaling pathway participates in sepsis. This increase in expression was reduced by MRS/MRM treatment, indicating that methane could inhibit the NLRP3/caspase-1/IL-1β signaling pathway to decrease pyroptosis offering a protective effect during sepsis.

In this study, we found that MRS reduced mortality and alleviated organ dysfunction by inhibiting cytokines, restraining oxidative stress and apoptosis, and downregulating NLRP3-mediated pyroptosis. This study proved that MRS mitigated sepsis and might be a new therapeutic approach for sepsis. However, these findings require more elaborate research and verification.

## CONCLUSION

In conclusion, the result of the present study demonstrated that MRS reduced mortality and alleviated organ dysfunction in sepsis. The mechanism of this promising effect could be that MRS inhibited proinflammatory cytokines and restrained oxidative stress and apoptosis, and downregulated NLRP3/caspase-1/ IL-1β signal pathway to decrease the pyroptosis. These finding suggested that MRS might be a potential therapeutic option in sepsis.

## MATERIALS AND METHODS

### Animals and cell culture

Wild-type C57BL/6 mice (male, 4-5 weeks old, weighing 20-25 g) were purchased from Animal Feeding Center of Xi’an Jiaotong University Health Science Center. All animal experiments complied by the guidelines of China Council on Animal Care and Use. In this study, the procedures of animal experiment were reviewed, approved, and supervised by the Institutional Animal Care and Use Committee of the Ethics Committee of Xi’an Jiaotong University Health Science Center, China.

RAW 264.7 macrophages were purchased from the cell Bank of Shanghai Institutes for Biological Science (Shanghai, China). RAW 264.7 cells were grown in DMEM supplemented with 10% heat-inactivated FBS and 100 U/mL penicillin, 100 mg/mL streptomycin, and cultured at 37°C with 5% CO2 in a humidiﬁed atmosphere.

To isolate peritoneal macrophages, C57BL/6 mice were injected with 1ml of 3% Brewer thioglycollate medium into the peritoneal cavity for 4 days. Then, 10 ml of PBS was administrated to the peritoneal cavity of the mouse, and peritoneal fluid was collected into a 50-ml tube and centrifuged to obtain the peritoneal exudate cells. Finally, the supernatant was removed, and cell pellet was resuspended in DMEM.

### Experimental design

This study was divided into two experiments. The aim of experiment 1 was to investigate the mechanisms underlying the protective effects of MRS on sepsis in vivo. Forty-five mice were randomly allocated into the following groups: (1) Sham control group (n = 9): C57BL/6 mice were given a sham laparotomy operation and the normal saline (NS) was administered at 2.5mL/kg every 12 h after the sham operation. (2) CLP+NS group (n = 9): C57BL/6 mice were given a laparotomy operation with cecal ligation and puncture (CLP) and the NS was administered at 2.5mL/kg every 12 h after CLP. (3) CLP+MRS (5 ml/kg) group (n = 9): C57BL/6 mice were given a laparotomy operation with CLP and the normal saline was administered at 5mL/kg every 12 h after CLP. (4) CLP+MRS (10 ml/kg) group (n = 9): C57BL/6 mice was given a laparotomy operation with CLP and the MRS were given at 10mL/kg every 12 h after CLP. (5) CLP+MRS (20 ml/kg) group (n = 9): C57BL/6 mice were given a laparotomy operation with CLP and the MRS was given at 20mL/kg every 12 h after CLP. The mice were observed for 5 days and recorded their survival rate. The other 36 mice were randomly divided into 3 groups described as below: (1) Sham control group (NS was administered at 2.5mL/kg 30 min and 12 h after sham operation, n = 12); (2) CLP + NS group (NS was administered at 2.5mL/kg 30 min and 12 h after CLP, n = 12); (3) CLP + MRS (MRS was administered at 10 ml/kg 30 min and 12 h after CLP, n =12). Mice were scarified 12 and 24 h after the surgery to collect blood and tissue samples.

The aim of experiment 2 was to investigate the mechanisms in a vitro experiment. RAW 264.7 and primary peritoneal macrophages were plated at a density of 1×10^7^ cells per 100-mm dish. The cells were rinsed with PBS and stimulated with LPS (1ug/ml) + ATP (5mM) to induce pyroptosis. (1) NM group (n = 3) was given normal medium. (2) MRM group (n = 3) was given methane-rich medium (MRM). (3) NM+L+A group (n = 3) was given normal medium with LPS and ATP. (4) MRM+L+A group (n = 3) was given methane-rich medium with LPS and ATP. The supernatants and proteins were collected 24 h after the stimulation of LPS+ATP.

### Methane-rich saline and methane-rich medium preparation 

Methane was dissolved in sealed normal saline and underwent high pressure (0.4 MPa) for 8 hours to produce MRS. Prepared MRS was stored in an aluminum bag under atmospheric pressure at 4 °C and sterilized by γ-radiation one day before utilization. Methane was dissolved into DMEM medium under 0.4 MPa pressure to produce methane-rich medium (MRM). The concentration of MRS and MRM was detected using gas chromatography (Shanghai Qiyang Standard Gas Ltd, Shanghai, China) as previous described [24]. According to calculation, the concentration of the MRS was 1.2 ~ 1.5 mmol/L, and the concentration of MRM was 1.4 ~ 1.8 mmol/L.

### Sepsis model

CLP surgery was performed as described below: along the midline of the abdomen, laparotomy was performed to isolate the cecum of mice after anesthetizing with 5% chloral hydrate. Using a 4-0 silk suture to ligate the almost 1/3 cecal tip. The cecum was separated with forceps and punctured twice with a 21-gauge needle [25] and squeezed to expel the cecal contents and placed back to the original location causing the bacteria enter the abdominal cavity. The mice were put back to their cages until completely recovery. The sham group were only incised abdomen, separated cecum and exposed for 5 minutes, then closed in layers [26].

### Histologic analysis

Twelve and twenty-four hours after CLP/sham operations, lung and intestine tissue samples were fixed in 4% paraformaldehyde for 48 h. Consecutive sections with 5 μm thickness were obtained from the paraffin. Hematoxylin and eosin (HE) staining of lung and intestine tissues were performed. The histological changes were assessed in a blind manner by two researchers using a light microscope, and a representative field was chosen for application. The lung was estimated by testing the W/D weight ratio. The superior and middle lobes of the right lung samples were weighed immediately after collection, then placed into an incubator at 80°C for 48 h to dry and weighed again. The lung W/D ratio was calculated by the wet weight divided by the dry weight [27]. Lung injury score was the sum of the individual score grades from 0, minimum; 1, mild; 2, moderate; 3, severe; and 4, maximum for each of the following 3 items: alveolar hemorrhage, infiltration or aggregation of inflammatory cells and thickness of the alveolar wall, ranging from 0 to 12 [28]. Intestine dysfunction was assessed by measuring the villus height in H&E staining [27]. Intestine injury score were the following: 0 = normal mucosa, 1 = development of subepithelial Gruenhagen's space and vacuolization at the villus tip, 2 = extension of the subepithelial space with moderate lifting of epithelial layer from the lamina propria, 3 = massive subepithelial lifting/sloughing and increased vacuolization from the tip to mid-portion of villi, 4 = epithelial lifting and vacuolization from the tip to lower portion of villi, and 5 = mucosal ulceration and disintegration of the lamina propria [29].

### Inflammatory cytokines assay

Twelve and twenty-four hours after the CLP/LPS+ATP, the blood samples and supernatants were collected to measure the levels of TNF-α and IL-6 using commercial ELISA kits (Dakewe, China) according to the manufacturer’s instructions.

### Immunohistochemical analysis

Twenty-four hours after the CLP operation, the lung and intestine tissues were harvested and processed for immunohistochemistry to estimate the activation of NF-κB, Caspase-1 and IL-1β. Briefly, the lung and intestine tissues were immersed into 4% paraformaldehyde to fix, embedded in paraffin and cut into sections 4 µm thick. The sections were deparaffinized with xylene, rehydrated with serial gradient ethanol. After incubation with 3% hydrogen peroxide for 15 min to block endogenous peroxidase activity, the sections were blocked with goat serum, and then incubated with primary antibodies against P65 (1:150; CST, USA) and Caspase-1 (1:150; Abcam, USA) and IL-1β (1:150; S Abcam, USA) overnight at 4°C. After those procedure, the sections were washed with PBS three times, and incubated with biotinylated secondary antibodies at room temperature for 40 min. Finally, the sections were incubated with diaminobenzidine tetrahydrochloride (DAB), counterstained with hematoxylin and mounted for microscopic examination.

### Reactive oxidative stress activity assay and DHE

The lung and intestine tissues were collected to measure the oxidative stress indexes 12 and 24 h after CLP. The levels of myeloperoxidase (MPO) and malondialdehyde (MDA) and the activities of glutathione (GSH) and superoxide dismutase (SOD) in lung and intestine tissues were tested by commercial biochemical kits (Nanjing Jiancheng, China) following the manufacturer’s instructions. For ROS detection, RAW 264.7 cells were incubated with Dihydroethidium (DHE) dye (sigma-aldrich, USA) for 30 min at a concentration of 3 μM. The sections were observed with a fluorescence microscope, and representative fields were chosen for application. The quantification of fluorescence intensity was performed using ImageJ software.

### Western blot assay

The protein expression in lung tissues and whole cells were detected by western blot. In brief, RIPA lysis buffer was used to extracted the total protein and nucleoprotein at 14000g for 15mins at 4°C. After the protein concentration was determined, the lysates were separated using sodium dodecyl sulfate-polyacrylamide gel electrophoresis (SDS-PAGE). Then the proteins were transferred onto polyvinylidene difluoride (PVDF) membranes. The resulting blots were blocked with 8% skim milk and incubated with anti-p65 antibody (1:1000; CST, USA), anti-p-p65 antibody (1:1000; CST, USA), anti-NLRP3 antibody (1:1500; Beyotime Biotechnology, China), anti-caspase-1 antibody (1:1000; Abcam, USA), anti-IL-1β antibody (1:1000; Abcam, USA), anti-Cytochrome-c antibody (1:1000; San Ying Biotechnology, China), anti-caspase-3 antibody (1:1000; CST, USA), anti-caspase-9 antibody(1:1000; Abcam, USA), anti-β-actin antibody (1:10000; San Ying Biotechnology ,China) overnight at 4°C. Subsequently, the bolts were washed three times with PBS and incubated with anti-rabbit and anti-mouse horseradish peroxidase-conjugated secondary antibodies (1: 10000; Abmart, China) for 1 h at 37°C. The proteins were detected with the chemiluminescence (ECL) system. The expression of proteins was normalized to β-actin as a reference.

### Pyroptosis assay

Pyroptosis was detected by propidium iodide (PI) staining following the manufacturer’s recommendations [30]. The sections were observed with a fluorescence microscope, and representative fields were chosen for application.

### Apoptosis assay

Twenty-four hours after CLP, tissue samples from lung and intestine were assessed with the transferase-mediated deoxyuridine triphosphate-biotin nick end labeling (TUNEL) staining with a fluorescence detection kit (In Situ Cell Death Detection Kit; Fluorescein, Roche, Switzerland). The sections were observed with a fluorescence microscope, and representative fields were chosen for application. Apoptotic cells were detected by Annexin 7AAD/PE Apoptosis Detection Kit (BD Biosciences) following the manufacturer’s recommendations. The cells were analyzed with a flow cytometry (ACEA Biosciences,Inc.). The sum of early and late apoptotic cell percentage was defined as apoptotic cell percentage.

### Immunofluorescence staining

Immunofluorescence staining for IL-1β expression in the RAW264.7 macrophages was performed as previous described. The primary antibody rabbit anti- IL-1β antibody (1:200, Abcam, USA) were incubated overnight at 4°. Then the second antibody goat anti-rabbit antibody (Servicebio, China, diluted 1:300) were incubated 60 min at room temperature and counterstained with 4’-6-diamidino-2-phenylindole (DAPI). The sections were observed with a fluorescence microscope, and representative fields were chosen for application.

### Statistical analysis

Values are presented as mean ± SD. All statistical analyses were performed by the SPSS18.0 software (SPSS Inc., Chicago, USA). For comparisons among multiple groups, one-way analysis of variance followed by the Student-Newman-Keuls post hoc test was used to determine significant differences. The figure was made by GraphPad Prism software (GraphPad Software, USA). All tests were two-sided and significance was accepted at p < 0.05.

### Ethics approval

All animal experiments complied by the guidelines of China Council on Animal Care and Use. In this study, the procedures of animal experiment were reviewed, approved, and supervised by the Institutional Animal Care and Use Committee of the Ethics Committee of Xi’an Jiaotong University Health Science Center, China.

### Availability of data and materials

The datasets used and/or analyzed during the present study are available from the corresponding author on reasonable request.
